# The evolution and genetic basis of a functionally critical skull bone, the parasphenoid, among Lake Malawi cichlids

**DOI:** 10.1093/evolinnean/kzae039

**Published:** 2024-12-05

**Authors:** Andrew J Conith, Sarah M Pascarella, Sylvie A Hope, R Craig Albertson

**Affiliations:** Department of Biological Sciences, DePaul University, Chicago, IL 60614, United States; Department of Biological Sciences, DePaul University, Chicago, IL 60614, United States; Biology Department, University of Massachusetts Amherst, Amherst, MA, 01002, United States; Biology Department, University of Massachusetts Amherst, Amherst, MA, 01002, United States

**Keywords:** adaptive radiation, craniofacial skeleton, genetic mapping, cichlid fishes

## Abstract

Adaptive radiation, whereby a clade pairs rapid speciation with rapid phenotypic evolution, can result in an uneven distribution of biodiversity across the Metazoan tree. The cichlid fishes of East Africa have undergone multiple adaptive radiations within the major rift lakes. Cichlid radiations are marked by divergence across distinct habitat gradients producing many morphological and behavioural adaptations. Here, we characterize the shape of the parasphenoid, a bone in the neurocranium that dissipates forces generated during feeding. We examine *Tropheops*, a group that has transitioned between deep and shallow habitats multiple times, to examine habitat-specific differences in parasphenoid shape. We find differences in the depth and length of the parasphenoid between *Tropheops* residing in each habitat, variation that may impact the ability of the cranium to resist force. We next use a hybrid cross between two cichlid species that differ in parasphenoid shape, *Labeotropheus* and *Tropheops*, to examine the genetic basis of these morphological differences. We perform genetic mapping and identify two genomic regions responsible for variation in parasphenoid shape. These regions are implicated in other functional traits including the oral jaws and neurocranium, indicating that the genetic landscape for adaptive evolution may be limited to a few loci with broad effects. Repurposing the same gene(s) for multiple traits via regulatory evolution may be sufficient for selection to drive transitions between habitats important for incipient stages of adaptive radiations.

## Introduction

Adaptive radiations can lead to bursts in phenotypic and/or taxonomic variation, and a variety of factors can precipitate such events (Schluter 2000, Losos 2010, Yoder *et al.* 2010), for example when a population moves into a novel environment with many available vacant niches (i.e. ecological opportunity), or when a trait(s) arises that provides access to previously inaccessible resources (i.e. key innovation). Ecological opportunity is thought to characterize diversification in lineages such as *Geospiza* finches in the Galápagos Islands (Rubin *et al.* 2022) and *Anolis* lizards in the Greater Antilles (Patton *et al.* 2021), while key innovations characterize diversification in other lineages such as the independent evolution of powered flight in birds, bats, and insects (Miller *et al*. 2023). In some cases, a lineage may diversify due to both ecological opportunity and key innovation such as in cichlids (Brawand *et al.* 2014), which encountered a wealth of ecological opportunities during the origin of the large rift lakes in East Africa and the evolution of a key innovation in the highly complex pharyngeal jaw (i.e. pharyngognathy). In these cases, adaptive radiation can lead to an incredible sum of biodiversity. Indeed, rift lake cichlids are one of the most species-rich vertebrate clades with more than 1500 species arising in a relatively short period of geological time (Rometsch *et al.* 2020).

Adaptive radiation in vertebrates can proceed simultaneously across different axes of variation (Streelman and Danley 2003), including traits tied to habitat (e.g. body shape/size, limbs/fins), feeding (e.g. jaws, teeth), and communication (e.g. coloration, behaviour, visual perception). However, given adaptive radiations occur so rapidly, ancestral state reconstruction becomes difficult to assess, meaning the order of these stages and their relative importance to shaping the trajectory of a given vertebrate radiation is difficult to discern without fossil evidence and can differ among closely related radiations (Richards *et al.* 2021). Identifying traits that will be most susceptible to phenotypic change as a population diverges will depend on which niches are vacant. Examining these functional traits can provide a window into how natural selection may act on a population to facilitate rapid filling of the available ecological space during an adaptive radiation.

As previously mentioned, cichlids from the rift lakes of east Africa reflect a dramatic example of an adaptive radiation that combines a clade experiencing ample ecological opportunity (e.g. the formation of the East African Rift System producing large, deep lakes) with an evolutionary key innovation (e.g. pharyngognathy, resulting in highly efficient food processing). There are multiple large lakes in this region (i.e. lakes Victoria, Tanganyika, and Malawi) and each has produced a distinct cichlid radiation (Muschick *et al.* 2014, Malinsky *et al.* 2018, Meier *et al.* 2023), alongside some smaller lakes (i.e. lakes Kivu, Edward, and Massoko; Malinsky *et al.* 2015, Meier *et al.* 2019). Notably, despite their independent invasion, the cichlid ecomorphs that arise in each lake converge upon strikingly similar morphologies due in part to the relative similarity in selective pressures (Albertson and Kocher 2006), alongside the combined roles of developmental and genetic constraints (Conith *et al.* 2021). At the genetic level, the ability of the rift lake cichlids to converge upon similar phenotypes is thought to result from a combination of introgression and selection on ancestral polymorphisms (Brawand *et al.* 2014, Masonick *et al*. 2023). The repeatability and predictability of the cichlid ecomorphs across lake systems appear to be occurring at both a phenotypic and a genetic level, and even within a lake system groups that transition between habitats (i.e. between deep and shallow) facilitate the evolution of a feeding apparatus that appears highly labile and can quickly respond to a population moving between deep and shallow habitats (McKaye and Marsh 1983, Cooper *et al.* 2010, Conith *et al.* 2020, Martinez *et al.* 2024).

**Figure 1.**
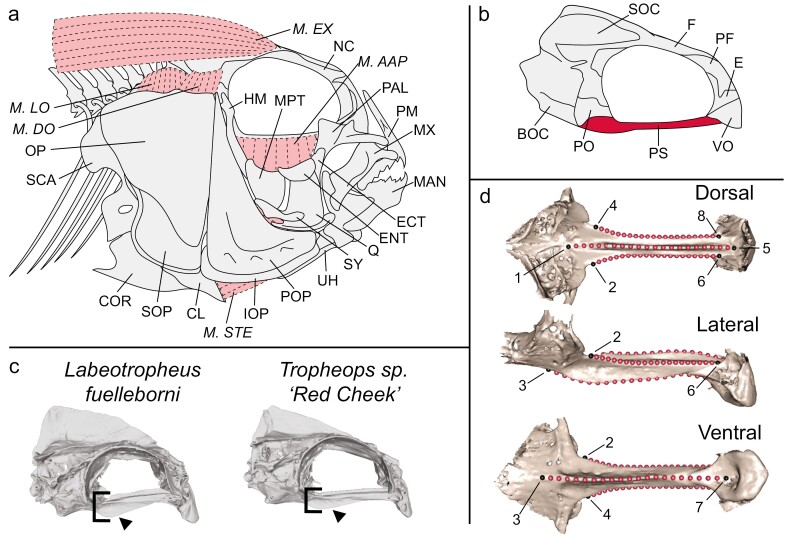
Overview of cichlid neurocranial anatomy and landmarking configuration used to characterize parasphenoid shape. All naming conventions follow Liem and Osse (1975). A, lateral aspect of the cranial bones after removal of lacrimal, adductor mandibulae muscles, orbital bones, and eyeball. Bones: CL, cleithrum; COR, coronoid; ECT, ectopterygoid; ENT, entopterygoid; HM, hyomandibular; IOP, interopercle; MAN, mandible; MPT, metapterygoid; MX, maxilla; NC, neurocranium; OP, opercular; PAL, palatine; PM, premaxilla; POP, preopercular; Q, quadrate; SCA, scapular; SOP, subopercular; SY, symplectic; UH, urohyal. Muscles: *M.AAP*, adductor arcus palatini; *M.EX*, epaxial muscle; *M.DO*, dilatator operculi muscle; *M.LO*, levator operculi muscle; *M.STE*, sternohyoideus muscle. B, lateral aspect of the neurocranium. BOC, basioccipital; E, ethmoid; F, frontal; PF, prefrontal; PO, prootic; PS, parasphenoid (red); SOC, supraoccipital crest; VO, vomer. C, representative *Labeotropheus* and *Tropheops* neurocrania models that illustrate the differences between an obligate algae scraper and algae nipper. Note the arrow depicting the ‘keel’ region of the parasphenoid bone, and the differences in height of the keel between taxa. D, landmark and semilandmark placement on a cichlid parasphenoid bone (black, fixed landmark; red, semilandmark).

Functional traits with clear links to either feeding or locomotion are probably among the most susceptible to change as a lineage diversifies to fill the available niche space during an adaptive radiation. These traits are often highly evolvable (i.e. ability to generate adaptive phenotypic variation), and can respond rapidly to selection (Pigliucci 2008, Payne and Wagner 2019). The cichlid neurocranium represents an interesting test-case to examine how a complex, multifunctional structure may change through the course of an adaptive radiation. The neurocranium exhibits extensive variation across species, which broadly aligns with foraging mode (Albertson and Kocher 2001, Cooper *et al.* 2011, Evans *et al.* 2017, 2021). However, the rate and magnitude of change do not build uniformly across the neurocranium, but rather this structure reflects a mosaic of traits that seem to evolve at different rates (Evans *et al.* 2019). In cichlids, much of the variation in the neurocranium is concentrated in the rostral region, which supports the feeding apparatus, while the caudal region is more constrained, probably due to its role in housing the brain (Conith *et al*. 2023). The biomechanical properties of the rostrum are governed in large part by the size and shape of the parasphenoid, a beam-like bone that runs between the eyes and connects the anterior tip of the neurocranium to the ventral base of the braincase (Fig. 1). The parasphenoid bone helps to resist compressive and torsional forces that propagate across the neurocranium during biting originating from the feeding apparatus (Cooper *et al.* 2011). The parasphenoid bone also serves as the origin for the adductor arcus palatini (AAP) muscle, which inserts onto the mesial sides of the hyomandibula, metapterygoid, and entopterygoid bones (Liem and Osse 1975). When contracted this muscle adducts the suspensorium to increase the volume of the buccal cavity during feeding. Thus, natural selection is expected to drive adaptive shape changes in the parasphenoid within those populations that experience large forces to reduce stress across the skull.

The cichlids of Lake Malawi are known to partition their habitat by depth and exhibit behavioural and morphological adaptations suited to this environmental gradient. Adaptations specific to deep or shallow habitats typically involve changes to the body and trophic morphology that permit more efficient exploitation of each niche (Cooper *et al.* 2010). Members of the *Tropheops* species complex represent a closely related group of rock-dwelling cichlids that are known to exploit both deep and shallow habitats and have transitioned between these different depth habitats multiple times (Ribbink *et al.* 1983, Conith *et al.* 2020). These populations differ in the degree of algal scraping behaviour, with those from shallow environments exhibiting lots of biomechanically demanding scraping behaviour, while those from deeper habitats exhibit some scraping, some shifting of detritus, and some feeding from the water column. Previous studies have demonstrated differences between *Tropheops* members from shallow or deep habitats within those bones involved in feeding such as the interopercle, mandible, lower pharyngeal jaw, maxilla, and pre-maxilla (Hu and Albertson 2014, Conith *et al.* 2020). These bones comprise aspects of the oral and pharyngeal jaws, alongside the suspensorium, but aspects of the neurocranium such as the parasphenoid have not been assessed across deep or shallow *Tropheops* members.

Here we characterize the shape of the parasphenoid bone from *Tropheops* members residing in deep and shallow habitats, and then use a hybrid mapping population between two cichlid taxa [*Tropheops* sp. ‘red cheek’ (some algal scaping behaviour) and *Labeotropheus fuelleborni* (obligate algal scaping behaviour)] to examine the genetic basis for shape differences in the parasphenoid that define the deep–shallow populations. We predict *Tropheops* from shallower environments will exhibit different shaped parasphenoid bones compared to those from deep environments, with the specific shape changes consistent with the ability to withstand higher forces (i.e. deeper-wider parasphenoid bones; Fig. 1C). Additionally, we expect that patterns of variation within our mapping population will parallel those among natural populations, providing an opportunity to dissect the genetic basis for this functionally important trait.

## Material and methods

### *Tropheops* specimens

We collected 65 individuals from the *Tropheops* species complex during two trips to the southern part of Lake Malawi in 1996 and 2001. In all, these individuals represented 15 species from 12 localities. The *Tropheops* sample contained representatives from across a broad depth gradient that we separated out into two distinct ecomorphs: deep (*N* = 26) and shallow (*N* = 39). Deep habitats are characterized by those environments that lie ~15 m beneath the water surface and are sheltered and rich in organic sediments that may coat algae on the rocks. Shallow habitats are the opposite, lying between 1 and 15 m from the surface and characterized by sediment-free environments that typically exhibited greater wave action (Ribbink *et al.* 1983, Albertson 2008). When possible, we collected multiple representatives for each species–locality designation, resulting in 27 total unique combinations from both deep (*N* = 9) and shallow (*N* = 18) environments.

We skeletonized our specimens using dermestid beetles and separated the neurocranium from the surrounding bones. We used a Phoenix V|tome|x S240 (General Electric) microcomputed tomography (μCT) scanner to obtain high-resolution 3D models of our *Tropheops* neurocrania. All specimens were scanned at 41–43 μm resolution at 80 kV and 300 μA. We exported image stacks using VGStudio v.2023.4 (Hexagon) and segmented the neurocrania using 3D Slicer (v.5.2.2, Fedorov *et al.* 2012). We saved the 3D models, then digitally dissected the parasphenoid bone from the neurocranium, removed noise, and reduced the mesh size using MeshLab (v.2023.12, Cignoni et al. 2008). Finally, we exported these final 3D models as ASCII ply files for subsequent morphometric analysis.

### Morphometric analysis

We placed a series of fixed and semilandmarks across the parasphenoid bone to characterize trophically important aspects of shape in our *Tropheops* species. In cross-section the parasphenoid resembles a plus symbol, so to best describe this shape we placed four fixed landmarks at the anterior ends of the parasphenoid on the dorsal, lateral, and ventral surfaces and mirrored that arrangement at the posterior end. Their placement on the outer edges of the parasphenoid ridges aims to capture the length, depth, and width of the parasphenoid bone. We connected the fixed landmarks on the dorsal, lateral, and ventral surfaces using four sets of 20 semilandmarks such that they lie on the ridges that span the parasphenoid (Fig. 1D).

All coordinate data from the fixed and semilandmarks were collected using Landmark v.3.6 (Wiley *et al.* 2005) and analysed using routines contained within the geomorph package v.4.0.8 in R v.4.4.1 (Baken *et al.* 2021). We used the digit.curves function from geomorph to uniformly arrange our four semilandmark sets across the ridges of the parasphenoid between two fixed landmarks.

We performed a Procrustes superimposition on our landmark data to remove the effects of size, translation, and rotation resulting in a series of landmark configurations that are in register (Rohlf 1998). We then calculated the mean shape configuration for each species–locality combination. To investigate the effects of allometry on our *Tropheops* shape data we performed a Procrustes ANOVA between parasphenoid centroid size and shape. We used a version of the Procrustes ANOVA, procD.pgls, that permits a correction for phylogenetic nonindependence (see the Phylogenetic comparative methods section for information on the tree) to assess allometry (Adams and Felice 2014). We did not find a significant effect of parasphenoid size on shape (*R*^2^ = .02, *Z* = 0.13, *P* = .467) and do not perform any correction for allometry. We also utilized the phylogenetically corrected Procrustes ANOVA method to examine evidence for parasphenoid shape differences between *Tropheops* species in deep and shallow habitats. Finally, we used a principal component analysis (PCA) to reduce our landmark dataset to a series of orthogonal axes that best explain variation in parasphenoid shape among *Tropheops* using the gm.prcomp function in geomorph. We extracted the scores from the first three principal components as together these three PCs explained almost 70% of the shape variation and would be used to compare the fit of various evolutionary models in a multivariate framework.

### Phylogenetic comparative methods

**Figure 2.**
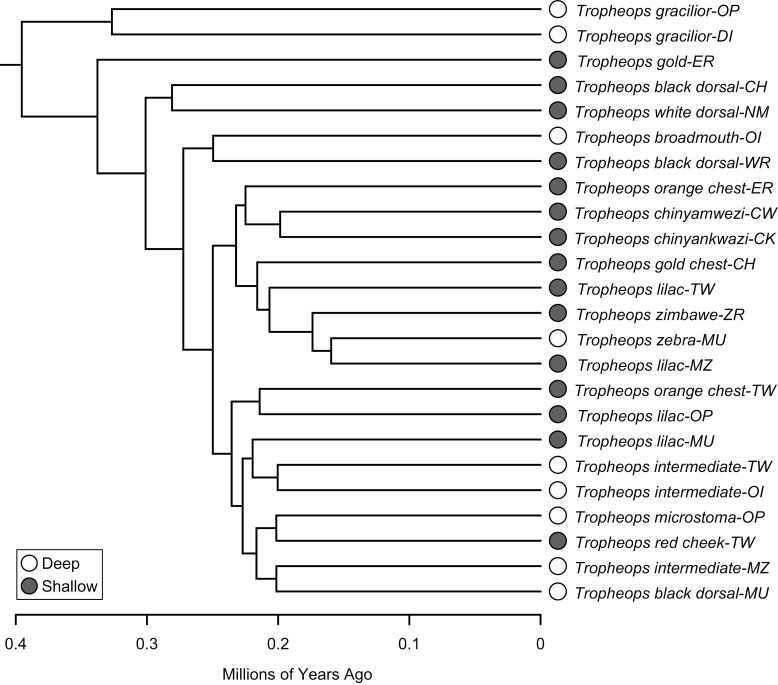
Tree depicting phylogenetic relationships of the *Tropheops* members used in this study. Phylogenetic tree taken from Conith *et al.* (2020) and constructed using AFLP markers. Colours denote occupation of either deep (white) or shallow habitats (grey).

To further examine how parasphenoid shape evolved, we used a previously constructed Bayesian time-calibrated tree of the *Tropheops* species complex that included members from across the southern part of Lake Malawi (Conith *et al.* 2020). This tree was built using amplified fragment length polymorphisms (AFLPs) and includes a total of 48 species, for which 33 are members of the *Tropheops* species complex. We obtained shape data for 27 members of the *Tropheops* species complex. Three of these taxa (*Tropheops* sp. ‘black dorsal’ and *Tropheops* sp. ‘intermediate’ both from Eccles Reef, alongside *Tropheops* sp. ‘orange chest’ from Mazinzi Reef) were not present in the phylogenetic tree and were dropped from all subsequent comparative assessments bringing the total to 24 species–locality combinations (Fig. 2).

To understand whether natural selection is driving parasphenoid shape toward two separate optima, based on habitat (shallow or deep) we fit various evolutionary models to our multivariate principal component parasphenoid shape data (Uyeda *et al*. 2015). We started by fitting two different single rate models to our parasphenoid principal component data using the mvBM and mvOU functions implemented in the R package mvMORPH (Clavel *et al*. 2015). The first model fitted a single rate Brownian motion (BM) model over the whole tree. Support for a BM model would suggest morphological variation is increasing uniformly over time. We then fit a single peak Ornstein–Uhlenbeck (OU) model to our parasphenoid shape data. Support for the OU model would suggest there is a single, optimal parasphenoid shape for all the *Tropheops* members. These models were compared to a multipeak OU model. The multipeak model contained two separate optima that corresponded to the major habitat types: deep and shallow. Support for the multipeak OU model would suggest natural selection is driving parasphenoid shape toward different optima based on habitat. For the multipeak OU model, we stochastically mapped changes in habitat depth onto the *Tropheops* tree using the Stochastic Mutational Mapping on Phylogenies (SIMMAP) tool (Bollback 2006) in Phytools (Revell 2012), and sampled 1000 character histories for habitat. This allowed us to incorporate evolutionary uncertainty in character histories to build a distribution of model parameters. To run the multipeak model, we again utilized the mvMORPH R package, and in the mvOU function set the model to ‘OUM’ which runs an OU model with different optima (θ) for each depth regime while holding the Brownian rate (σ^2^) and strength of selection (α) constant. We report the median and 95% confidence intervals for our multipeak OU evolutionary models. We obtained depth regime information from previously published ecological surveys of Lake Malawi (Ribbink *et al.* 1983).

Given the tendency for phylogenetic trees built from AFLP data to exhibit long terminal branches and short internal branches because of the restriction input data, we applied branch scaling methods to examine if the results from our Procrustes ANOVA and evolutionary model fitting analyses were robust to variation in branch length. We transformed our tree using Pagel’s lambda (λ); λ is a scaling parameter based on the association between taxa (Pagel 1999). We produced an additional tree with adjusted branch lengths by scaling our original tree to λ = 1.5, which has the effect of extending the internal branches and shortening the external branches. We rescaled our tree using the rescaleRR R function in the RRphylo library (Castiglione *et al.* 2021).

To account for small sample sizes in our model fitting procedure we used the second-order Akaike Information Criterion (AICc) to select from among the best models. The fit of a model is significantly better than any other model if the difference in AICc score is greater than two units and substantially better if greater than four units (Burnham and Anderson 2002).

### Quantitative loci mapping

To build our genetic map for analysis of quantitative trait loci (QTL) we crossed a wild-caught *Labeotropheus fuelleborni* (LF) female from Makanjila Point with a *Tropheops* sp. ‘red cheek’ (TRC) male from Chizumulu Island. LF are obligate algal scrapers that use their large robust jaws to pry algae from the rocks and exhibit tall parasphenoid bones. TRC are also algal foragers that employ a nip and twist-like motion to pull strands of attached algae from rocks and exhibit a relatively short parasphenoid bone (Ribbink *et al.* 1983). We further crossed the full-sibling F_1_ family to produce an F_2_ population and then performed further intercrosses by randomly interbreeding individuals from different families up to the F_5_ generation (*N* = 636). While mapping studies can use an F_2_ population for QTL, intercrossing to the F_5_ generation permits more recombination events and increases the resolution of mapping intervals. We took hybrid tissues from caudal fin clips of all 636 F_5_ hybrids and then extracted genomic DNA following standard RAD-seq (restriction-site associated DNA sequencing) procedures (Chutimanitsakun *et al.* 2011). The F_5_ hybrid population did not exhibit a tractable pattern of Mendelian inheritance and so a map could not be built using standard linkage mapping. We therefore used a genetic map derived from the F_2_ generation of the same pedigree and matched our F_5_ hybrids to this map (Albertson *et al.* 2014). We genotyped a subset of 812 evenly spaced genetic markers in the F_5_ population to perform future QTL analyses.

Following construction of the map, we scanned 413 F_5_ hybrids using an X-Tek HMXST 225 μCT device (Nikon). All scans were acquired at ~30 μm resolution using 80–125 kV and 75–120 μA. We used VGStudio v.2023.4 (Hexagon) to extract Z-stack images from the X-ray images and segmented the hard tissues using Mimics (v.19 Materialise NV), before exporting the 3D models to Geomagic 2014 (v.1.0 3D Systems). We used Geomagic (v.2014.1) to digitally dissect the parasphenoid from the rest of the neurocranium to make the placement of 3D landmarks more simplistic. The landmarking scheme followed that used in the wild-caught *Tropheops* population described above (see Morphometric analysis). In the hybrid shape data there is an association between size and shape (*R*^2^ = .01, *Z* = 3.74, *P* = .001), but given the low amount of variation explained (~1%), and to remain consistent with the *Tropheops* analysis, we do not correct the hybrid shape configurations for allometry.

Again, we perform a PCA to reduce our hybrid shape data to a series of axes that best explain variation in the parasphenoid. PC1 explained a substantial amount of variation (24.94%) in the hybrids and the primary axis of variation was similar between the *Tropheops* and hybrid populations. Both populations varied along an axis that reflected changes in parasphenoid depth (see Results for more information). As a result, we extracted this parasphenoid depth PC to further examine the genetic basis via QTL analysis.

We used multiple QTL mapping (MQM) methods implemented in r/qtl (Broman *et al.* 2003, Broman and Sen 2009, Arends *et al.* 2010) to determine which regions of the genome may be responsible for variation in parasphenoid depth. We searched for putative loci by first performing a QTL scan using the scanone function to uncover QTL peaks that reflect positions where genotype–phenotype associations are strong. We sequentially added more markers as cofactors to this initial model based on the position of peaks in our initial scans. We then performed an MQM analysis via the mqmscan function. MQM determined the fit of these cofactors through maximum-likelihood backward elimination. More cofactors were added to the model until we maximized the logarithm of odds (LOD) score. To identify significant peaks from our MQM scan we utilized the mqmpermutation function. This function shuffles the phenotype data relative to the genotype data to generate a null distribution of LOD scores at each marker based on 1000 permutations. If a QTL marker exceeds the permuted LOD score at a 5% threshold (indicating 95% of the permuted scores fall below that threshold) then it is deemed significant. To determine the size of the genomic region around a significant QTL we calculated an approximate Bayesian credible interval using the bayesint function. Within this interval, a prospective candidate gene that impacts parasphenoid depth should reside.

We identified two major peaks from our QTL, linkage group (LG) 7 and 13 and built a fine map to investigate the association between genotype and phenotype using a more densely constructed map that contained one marker every ~490 kb. Fine mapping with greater marker cover results in higher resolution and the ability to refine the initial intervals to a smaller number of base pairs for which the candidate gene(s) should reside. We identified additional RAD-seq single nucleotide polymorphisms (SNPs) in the F_5_ population and used them to map across the whole of LG7 and LG13. To build the fine maps, we used the used the *Maylandia zebra* (MZ) genome (UMD2a; GCA_000238955.5) to anchor QTL intervals to specific stretches of physical sequence along both LG7 and LG13 (Conte and Kocher 2015). We pulled additional markers from our original RAD-seq dataset for LG7 and LG13 and used VCFtools (v.0.1.16) to create smaller maps specific to each LG (Danecek *et al.* 2011). We used the vcfR (v.1.10.0) package in R to recode the VCF files into a format that is readable by r/qtl such that it can readily extract the genotypic information at each marker for each hybrid (Knaus and Grünwald 2017). For both the LG7 and LG13 maps we assessed the difference in average trait values at each marker between those F_5_ hybrids that are homozygous for the LF allele and those that are homozygous for the TRC allele using the effectsplot function in r/qtl.

Finally, we examined the extent of genetic divergence across LG7 and LG13 to uncover possible signatures of selection at several markers. Specifically, we compared wild-caught LF (*N* = 20) and TRC (*N* = 20) populations to identify regions in the genome that appear to be diverging toward distinct alleles using F statistic estimates (*F*_ST_) at each locus. An *F*_ST_ value close to 1.0 implies large differentiation among populations at a given locus (Nei 1987). We Z-transformed all our *F*_ST_ values (*zF*_ST_) from across the genome to gather a series of thresholds for genetic divergence between our parental populations. We plotted two thresholds (*Z* = 2; *Z* = 1) to demonstrate the degree of divergence at each marker on our fine maps. Between the LF and TRC populations, a *Z*-score of 1 was broadly equivalent to an *F*_ST_ score of ~0.6, which is a value that has previously been used to indicate significant divergence between populations (Mims *et al.* 2010).

## Results

### Morphological diversification in parasphenoid shape

**Figure 3.**
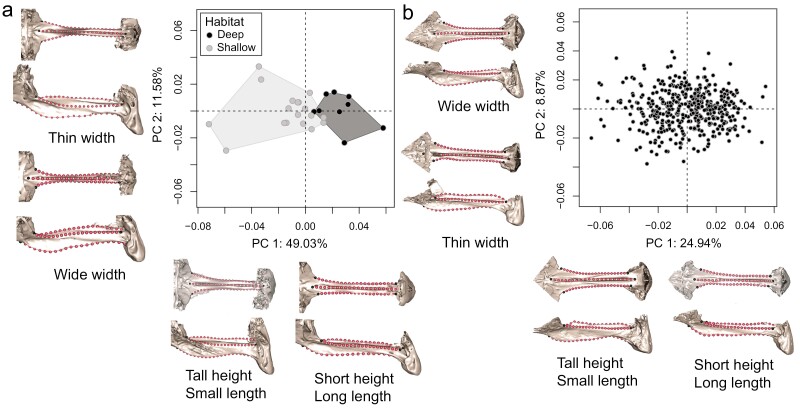
Principal component morphospace plots for the *Tropheops* and F_5_ LF × TRC hybrid parasphenoid bone shape data. Example parasphenoid bones for each end of the PC axes are illustrated using models with landmarks mapped onto the mesh. A, wild caught *Tropheops* parasphenoid bone morphospace (black point, deep *Tropheops* ecomorph; grey point, shallow *Tropheops* ecomorph). B, F_5_ LF × TRC hybrid parasphenoid bone morphospace.

*Tropheops* taxa residing at different lake depths appear to exhibit broad similarity in their parasphenoid shapes (*R*^2^ = .11, *F*_(1,22)_ = 2.83, *P* = .20) based on our full-shape Procrustes ANOVA (Fig. 3A; Supporting Information Table S1). When we examine the morphospace PC1 (49.03% of the variation) was explained by differences in parasphenoid length and height, and distinguished shallow from deep *Tropheops*. Indeed, a phylogenetic ANOVA on the PC1 scores indicated strong support for differences in PC1 scores between depths (*R*^2^ = .46, *F*_(1,22)_ = 18.50, *P* < .001). *Tropheops* from shallow habitats typically exhibited relatively short and stout (e.g. in the lateral view) parasphenoid bones, while those from deep habitats possessed on average long and slender parasphenoids. PC2 (11.58% of the variation) described differences in parasphenoid width, but there was substantial overlap between deep and shallow *Tropheops* taxa, indicating that width does not vary by habitat depth in this lineage. A phylogenetic ANOVA on the PC2 scores confirmed the lack of separation between depths (*R*^2^ = .007, *F*_(1,22)_ = 0.16, *P* = .68). PC3 (8.46% of the variation) reflected differences in the profile of the parasphenoid ‘keel’ and did not differ by habitat (*R*^2^ = .03, *F*_(1,22)_ = 0.72, *P* = .398). We note that all the phylogenetic ANOVA results listed above are robust to scaling branch lengths to force terminal branches to become shorter and internal branches to be longer (Table S2).

The multivariate evolutionary model analysis on PCs 1–3 favoured a multipeak OU model with separate optima for those taxa residing in shallow or deep habitats (Table 1). The two-peak OU model is 2.5 AICc units away from the next competing evolutionary model. Natural selection is therefore probably driving the parasphenoid toward different shape optima based on habitat, with shallow dwelling *Tropheops* exhibiting short/stout parasphenoid bones compared to deeper dwelling *Tropheops* species. Additionally, these results do not appreciably change when we scale the internal and external branches of our tree (Supporting Information Table S3).

**Table 1.** Fit of multivariate Brownian and OU evolutionary models to parasphenoid bone shape PCs.

Model	Log likelihood	AICc	Delta AICc	AICc weight
Ornstein–Uhlenbeck MP	212.01 (211.94, 212.09)	−375.12 (−375.26, −374.97)	0	0.77
Brownian motion	196.76	−372.63	2.49	0.22
Ornstein–Uhlenbeck SP	202.29	−366.02	9.10	<0.01

Models are ranked from best to worst based on AICc weights. Two OU models were fit, one multipeak model that permitted separate parasphenoid shape optima for the two habitat types: deep and shallow [Ornstein–Uhlenbeck (MP), multipeak], and a single-peak model that permitted only a single optimum [Ornstein–Uhlenbeck (SP), single peak]. Values in parentheses are 95% confidence intervals.

### Hybrids recapitulate evolved variation

The range of parasphenoid morphologies present in our hybrid cross comprised a spectrum from more LF-like to more TRC-like phenotypes. The primary axis of variation in the LF × TRC F_5_ hybrids matches the primary axis of variation in our sample of *Tropheops* from across the depth gradient; hybrids differ in the height and length of their parasphenoid bones (Fig. 3B; Supporting Information Table S4). PC1 (24.94% of the variation) describes differences in parasphenoid height and length, while PC2 (8.87% of the variation) explains differences in width. Notably, both of these axes recapitulate the major changes we see occurring across our *Tropheops* sample.

**Figure 4.**
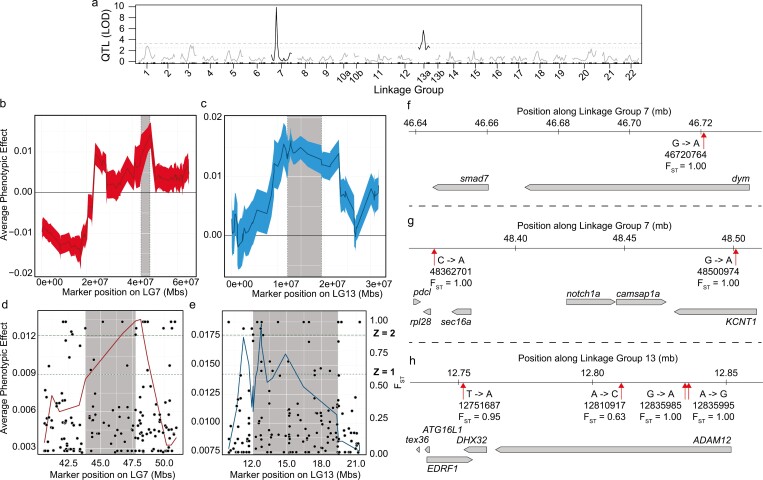
QTL mapping and fine mapping of PC1 (parasphenoid height and length) of the F_5_ LF × TRC hybrids and genetic regions of interest with the greatest segregation between *Labeotropheus* and *Tropheops* populations. A, genome MQM scan illustrating the peak genotype–phenotypic effects are localized to LG7 and LG13a. B, fine map for PC1 on LG7 (dark red solid line, mean; light red thick line, standard error). C, fine map for PC1 on LG13 (dark blue solid line, mean; light blue thick line, standard error). D, fine map for PC1 on LG7 centred around the phenotypic effect peak with *F*_ST_ markers mapped on. E, fine map for PC1 on LG13 centred around the phenotypic effect peak with *F*_ST_ markers mapped on. F, gene track for the phenotypic effect peak centred on *smad7* and *dym* along LG7. G, gene track for the phenotypic effect peak centred on *notch1a* along LG7. H, gene track for the phenotypic effect peak centred on *ADAM12* along LG13. Nucleotide annotations describe changes from TRC to LF at a given marker.

Given PC1 describes changes in parasphenoid shape that differentiate deep- from shallow-dwelling *Tropheops* species (i.e. length/height), we extracted hybrid PC1 scores for QTL mapping. Our QTL analysis identified two primary regions in the genome that are associated with variation in parasphenoid length/height (Supporting Information Table S5): LG7 and LG13 (Fig. 4A). The Bayesian credible intervals revealed a region 8.92 cM long between 19.12 and 28.04  cM on LG7 and another region 23.21 cM long between 0.77 and 23.98 cM on LG13. The LOD scores for both LG7 (9.94 LOD) and LG13 (5.73 LOD) were above the 5% genome-wide LOD threshold level derived from permutation tests (5% = 3.39 LOD; 10% = 2.77 LOD). The QTL on LG7 explained 28.37% of the phenotypic variation in the F_5_ hybrids, and the QTL on LG13 explained 16.18% of the variation. Modes of inheritance reflected dominant and additive effects, on LGs 7 and 13, respectively.

Our fine mapping results showed peaks within the Bayesian credible intervals for both LG7 and LG13 (Fig. 4B, C). For LG7, the interval spanned from 43.8 to 47.8 Mb, and the peak, whereby the differences in the average phenotypic effect are greatest, resided at 47.75 Mb. For LG13, the interval spanned from 12.1 to 19.4 Mb and the peak was at 12.84 Mb.

We further investigated regions that exhibited both high phenotypic effects (i.e. large differences between hybrids exhibiting LF or TRC genotypes at a given marker), and high *F*_ST_ (i.e. *F*_ST_ ≈ 1.0) scores (Fig. 4D, E; Supporting Information Table S6), derived from comparing natural populations of LF and TRC. For LG7, we identified six *F*_ST_ scores with values greater than 0.9 within a 2-Mb region surrounding the peak phenotypic effects (Fig. 4F, G). Two were located near *PLPP1*, *mtrex*, and *DHX29* (LG7: 47752984; LG7: 47802408), which are unlikely to control variation in bone shape (Parsyan *et al.* 2009, Fuchs *et al.* 2022, Yu *et al.* 2023); however, there were three adjacent to *notch1a* (LG7: 48362701; LG7: 48500974; LG7: 48709738), and one that was near *smad7* and *dymeclin* (LG7: 46720764). *Notch1a*, *smad7*, and *dymeclin* are known to exhibit expression differences between *Labeotropheus* and *Tropheops* in oral and pharyngeal jaw bone tissues (Conith and Albertson 2021), and therefore represent strong candidate genes. For LG13, we found three high *F*_ST_ scores that correspond to our largest average phenotypic effect peak (LG13: 12751687; LG13: 12835985; LG13: 12835995). Two of these *F*_ST_ scores reside within intronic regions of *ADAM12* and the other sits around 12 kb from the 3ʹ untranslated region (3ʹUTR) of the same gene (Fig. 4H). *ADAM12* was previously implicated in controlling ligament morphology differences between *Labeotropheus* and *Tropheops* (Conith *et al.* 2018), and represents another robust candidate gene.

## Discussion

### Functional implications of parasphenoid shape variation

The parasphenoid is a functionally important bone that bolsters the base of the rostral portion of the skull, while providing a site for some feeding muscles to attach. Here we show that this bone varies among cichlid populations that reside in either deep or shallow habitats. Populations of the algal-foraging *Tropheops* have transitioned between deep and shallow environments multiple times (Conith *et al.* 2020), and natural selection appears to be driving the shape of the parasphenoid toward two different habitat-specific optima. In shallow environments the parasphenoid bones are short and stout, while in deep environments the parasphenoid bones are long and gracile. Variation in parasphenoid shape is reflective of dietary differences between the deep and shallow *Tropheops* populations, with deeper members typically feeding using a greater range of behaviours (i.e.i.e. sifting, suction, scaping) relative to the shallow members with fewer behaviours. These dietary differences probably arise because of the environmental differences between depths. *Tropheops* species in deeper habitats encounter more sediment-rich and sheltered environments whereby their feeding apparatus must sift through these sediments prior to nipping algae. Those in shallower habitats encounter sediment-free, wave-swept environments with a feeding apparatus ready for the mechanically demanding task of scraping filamentous algae straight from the rocky substrate (Ribbink *et al.* 1983, Albertson 2008). Shallow *Tropheops* populations gather most of their food by scaping/nipping algae off rocks and the shape of the parasphenoid bone can have large implications for how the forces produced by these scraping behaviours are distributed through the parasphenoid and across the skull. Indeed, the greater height and shorter length of the parasphenoid bone in shallow populations probably aids the dispersion of forces across the skull, as demonstrated by a comparative finite element study among cichlids that employ either a biting/scraping (i.e.i.e. *L. fuelleborni*) or suction (i.e.i.e. *Maylandia zebra*) mode of feeding (Cooper *et al.* 2011). Additionally, shallow populations exhibit large parasphenoid ‘keels’ (see Fig. 1) that probably strengthen the connection between the rostral region of the neurocranium and the ventral portion of the braincase (prootic), thus mitigating a build-up of stress at the connection between the parasphenoid and braincase. Notably, the patterns observed here match those in other *Tropheops* bones more directly associated with feeding, including the mandible, premaxilla, maxilla, and pharyngeal jaw (Conith *et al.* 2020), suggesting that a common axis of selection is coordinating change across multiple bones. In all cases natural selection is probably driving differences between habitats in traits associated with either generating/resisting force or generating speed.

### QTL hotspots and coordinated evolution across functionally related bones

Our genetic mapping cross between *L. fuelleborni* and *Tropheops* sp. ‘red cheek’ produced a range of parasphenoid bone shapes, and recapitulated variation observed among natural populations. Mapping the first principal component, which captured the stout to gracile morphology axis, revealed two genomic regions of interest. These regions are associated with those aspects of parasphenoid shape that define the differences between deep and shallow *Tropheops* populations. The parasphenoid shape QTL on LG7 had the largest effect, indicating a gene or suite of genes in this region have an outsized role in producing the shape differences between populations. In cichlids, this region of LG7 has appeared in multiple QTL analyses, in different crosses, and for vastly different tissue types. For example, QTL for phenotypic variation in oral and pharyngeal jaws (Albertson *et al*. 2003, 2005, Conith and Albertson 2021), teeth (Albertson *et al.* 2003), scales (Albertson *et al.* 2018), hearts (Conith *et al.* 2021), fins (Navon *et al.* 2021), gill rakers (Zogbaum *et al*. 2021), brains (Conith *et al.* 2023), ligaments (Conith *et al.* 2018), coloration (Albertson *et al.* 2018), and body shape (DeLorenzo *et al.* 2023) have all mapped to this region. We also recorded a QTL on LG13 for parasphenoid shape. While not arising as commonly as LG7, QTL hits on LG13 were reported for phenotypic variation in ligament (Conith *et al.* 2018), cleithrum (Conith *et al.* 2021), and fin morphology (DeLorenzo *et al.* 2023). QTL can sometimes cluster together, particularly when trait function, development, and/or regulation are correlated, and are referred to as QTL ‘hotspots’ (Martin and Orgogozo 2013).

QTL hotspots may arise due to pleiotropy, genetic linkage, or high allelic polymorphism (Martin and Orgogozo 2013, Wu *et al*. 2021). The presence of QTL hotspots suggests a relatively small genomic region can control a wealth of functionally important phenotypic variation, and these regions could provide a source of genetic variation that may permit the rapid radiation and repeated transitions between habitats observed in cichlid populations (i.e. *Tropheops*) and other vertebrate radiations (Golcher-Benavides and Wagner 2019, Archambeault *et al.* 2020, Gillespie *et al.* 2020, Feller and Seehausen 2022). Such genomic intervals can arise via ancestral hybridization events whereby introgression and recombination produce a series of large insertion or deletion polymorphisms (McGee *et al.* 2020, Svardal *et al.* 2020). While these types of polymorphisms have been shown to be specific to cichlids with particular ecologies (McGee *et al.* 2020), it remains to be seen whether they co-localized with QTL hotspots or contain co-adapted gene sets that may facilitate ecological shifts.

### Candidate genes for parasphenoid shape variation

The peaks from our QTL analysis reside close to candidate genes that were previously implicated in controlling functionally important variation in the cichlid head (Conith *et al.* 2018, 2021, Conith and Albertson 2021). On LG7, our fine mapping peak and high *F*_ST_ values, indicating large differences in phenotype between hybrids and alternate fixation of markers between populations, is close to three genes that have the capacity to control differences in parasphenoid bone shape: *smad7*, *dym*, and *notch1a*. *Dym* encodes a membrane protein that organizes the Golgi apparatus and is essential in endochondral bone formation and brain development (Osipovich *et al.* 2008). Mutant models show abnormal size and shape of cranial bones due to dysregulated chondrocyte development producing stunted brain development microcephaly in humans (Denais *et al.* 2011). *Smad7* inhibits TGF-β, Nodal, BMP, and Activin signalling pathways, and is known to inhibit endochondral bone formation by limiting osteogenesis and can also enhance bone resorption (Li *et al.* 2014, Wu *et al.* 2024). *Notch1a* encodes a receptor involved in intercellular signalling and can bind ligands on neighbouring cells. The Notch1a receptor is involved in bone development and homeostasis and plays a key role in coordinating craniofacial embryogenesis (Pakvasa *et al.* 2021). On LG13 our phenotypic effect peak and high *F*_ST_ markers reside in and around *ADAM12*. ADAM12 is a transmembrane protein important in cell adhesion and intracellular signalling (Kveiborg *et al.* 2008) and plays an important role in bone growth through regulating chondrocyte proliferation (Kveiborg *et al.* 2006). In cichlids, *ADAM12* was previously demonstrated to be important in regulating ligament growth (Conith *et al.* 2018), illustrating the diverse role this transmembrane protein plays across multiple tissue types. There is some evidence to suggest that expression of some ADAM members is regulated by Notch signalling (Groot and Vooijs 2012, Zhou *et al.* 2022), which suggests epistatic effects may be present between loci on LG7 and LG13, and epistatic effects have the capacity to drive morphological divergence (Bourg *et al.* 2024).

## Conclusions

Lineages undergoing adaptive radiation pair high rates of speciation with rapid morphological diversification, but understanding why there are differences in the propensity to radiate and how these differences arise involves understanding the genetic landscape in concert with the ecological pressures that lineages may experience. Lake Malawi cichlids represent a lineage undergoing an adaptive radiation for the last 1–2 Myr (Seehausen *et al.* 2008). As is common in the other rift lakes, the radiation of Lake Malawi cichlids has involved the repeated invasion of deep- and shallow-water environments each radiation with a specific combination of size, jaw, tail, and head shape traits (Cooper *et al.* 2010, DeLorenzo *et al.* 2023). Here we examined the phenotypic consequences of this habitat partitioning on a functionally important bone in the neurocranium, the parasphenoid, in an evolutionarily rich species-complex, *Tropheops*. Using a genetic mapping approach, we identified two genomic regions that were associated with differences in parasphenoid shape, and this phenotypic variation in parasphenoid shape defined the differences between cichlids living in deep vs shallow habitats. These regions have arisen in other genomic scans using other functionally relevant traits such as the jaws (Conith and Albertson 2021) and neurocranium (Conith *et al.* 2023), constituting QTL ‘hotspots’. This suggests that the genetic landscape for rapid change in feeding anatomy may be rather limited and that transitions to new niches, or between previously colonized niches, may be facilitated by selection calling on few loci with broad effects (Chan *et al.* 2010, Marques *et al.* 2022). These genes can be deployed again and again, tweaked by a variable regulatory landscape, to alter different traits with the same tissue types, or similar tissues in different locations along the body. Those populations that are best able to exploit this continued repurposing of genes should be able to readily generate adaptive variation and be highly evolvable (i.e. Pigliucci 2008, Payne and Wagner 2019). Thus, a focus on regions of the genome with effects on suites of traits should be a fruitful and important line of future research, especially in lineages undergoing rapid adaptive radiation.

## Supplementary Material

kzae039_suppl_Supplementary_Tables

## Data Availability

The data underlying this article are available on GitHub and can be accessed through github.com/andrewjohnconith/Parasphenoid.

## CRediT Statement

AJC and RCA devised the study, SMP and SAH performed morphometric analysis, and AJC performed statistical genetic analyses. All authors wrote, read, and approved the final version of the manuscript.
